# D-Cysteine Activates Chaperone-Mediated Autophagy in Cerebellar Purkinje Cells via the Generation of Hydrogen Sulfide and Nrf2 Activation

**DOI:** 10.3390/cells11071230

**Published:** 2022-04-05

**Authors:** Erika Ueda, Tomoko Ohta, Ayumu Konno, Hirokazu Hirai, Yuki Kurauchi, Hiroshi Katsuki, Takahiro Seki

**Affiliations:** 1 Department of Chemico-Pharmacological Sciences, Graduate School of Pharmaceutical Sciences, Kumamoto University, Kumamoto 862-0973, Japan; 219y1012@st.kumamoto-u.ac.jp (E.U.); tm.svki@gmail.com (T.O.); kurauchy@kumamoto-u.ac.jp (Y.K.); hkatsuki@gpo.kumamoto-u.ac.jp (H.K.); 2 Department of Neurophysiology & Neural Repair, Graduate School of Medicine, Gunma University, Maebashi 371-8511, Japan; konnoa@gunma-u.ac.jp (A.K.); hirai@gunma-u.ac.jp (H.H.)

**Keywords:** aromatic-turmerone, dopaminergic neurons, microglia, Nrf2

## Abstract

Chaperone-mediated autophagy (CMA) is a pathway in the autophagy-lysosome protein degradation system. CMA impairment has been implicated to play a role in spinocerebellar ataxia (SCA) pathogenesis. D-cysteine is metabolized by D-amino acid oxidase (DAO), leading to hydrogen sulfide generation in the cerebellum. Although D-cysteine alleviates the disease phenotypes in SCA-model mice, it remains unknown how hydrogen sulfide derived from D-cysteine exerts this effect. In the present study, we investigated the effects of D-cysteine and hydrogen sulfide on CMA activity using a CMA activity marker that we have established. D-cysteine activated CMA in Purkinje cells (PCs) of primary cerebellar cultures where DAO was expressed, while it failed to activate CMA in DAO-deficient AD293 cells. In contrast, Na_2_S, a hydrogen sulfide donor, activated CMA in both PCs and AD293 cells. Nuclear factor erythroid 2-related factor 2 (Nrf2) is known to be activated by hydrogen sulfide and regulate CMA activity. An Nrf2 inhibitor, ML385, prevented CMA activation triggered by D-cysteine and Na_2_S. Additionally, long-term treatment with D-cysteine increased the amounts of Nrf2 and LAMP2A, a CMA-related protein, in the mouse cerebellum. These findings suggest that hydrogen sulfide derived from D-cysteine enhances CMA activity via Nrf2 activation.

## 1. Introduction

Hydrogen sulfide has recently been gaining significance as a novel gas mediator [1]. It is endogenously generated from L-cysteine and triggers polysulfation of cysteine residues and glutathione, leading to alteration in protein functions and increased antioxidative activity [2]. Hydrogen sulfide is known to protect various tissues, including those of the brain and liver, from toxic stimuli. However, hydrogen sulfide and its nonselective donors would induce multiple side effects by modifying various protein functions [2]. Therefore, the development of tissue-specific donors is required for the therapeutic application of hydrogen sulfide.

Spinocerebellar ataxia (SCA) is a group of autosomal dominant neurodegenerative diseases characterized by progressive cerebellar ataxia and cerebellar atrophy [3,4]. Although SCA is classified as SCA type 1 (SCA1) to type 48 (SCA48) based on the differences in causal genes, the common functions of these SCA-causing genes are still unidentified. Therefore, the common pathogenic mechanisms of SCAs remain unclear. We have previously demonstrated that various SCA-causing proteins commonly induce improper dendritic development in primary cultured cerebellar Purkinje cells (PCs) [5,6,7], which are the sole output neurons from the cerebellar cortex and are characterized by highly developed dendrites [8]. Additionally, the expression of SCA-causing proteins in cerebellar neurons commonly triggers early glial activation before cerebellar neurodegeneration in mice [6,7,9]. These in vitro and in vivo phenotypes can be helpful in identifying pathogenic mechanisms and developing novel therapeutics or preventives commonly for various SCA types.

**Figure 1:**
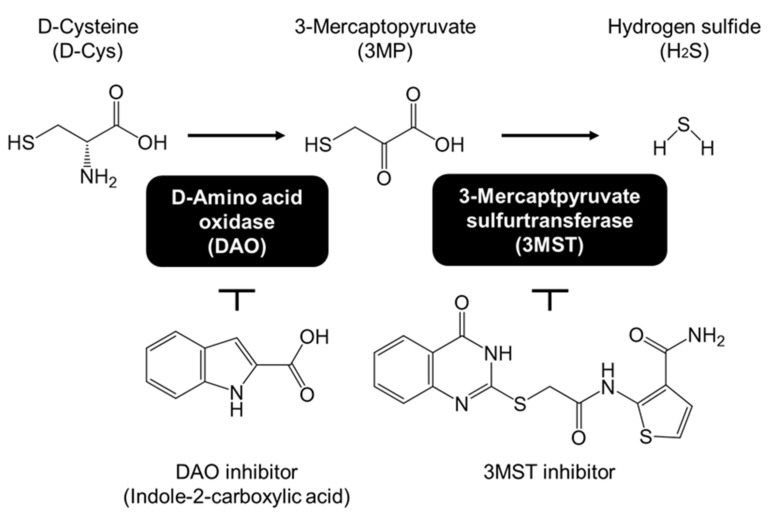
Pathway of hydrogen sulfide generation from D-cysteine with the chemical structures of inhibitors.

We recently reported that D-cysteine ameliorates impaired dendritic growth in primary cultured PCs expressing several SCA-causing proteins [7]. Additionally, long-term treatment with D-cysteine prevents the onsets of motor impairment and glial activation in SCA1-model mice [7]. Although the roles of D-cysteine are not yet fully elucidated in mammals, it is known to be converted into 3-mercaptopyruvate (3MP) by D-amino acid oxidase (DAO), followed by hydrogen sulfide production from 3MP by 3-mercaptopyruvate sulfurtransferase (3MST) (Figure 1) [10]. In mammals, DAO is found to be localized specifically within the cerebellum, liver and kidney [11,12]. Therefore, we postulate that D-cysteine could be the selective hydrogen sulfide donor for these tissues. Additionally, we have previously reported that D-cysteine promotes dendritic development in cultured PCs via hydrogen sulfide production [13]. Therefore, the preventive effect of D-cysteine is considered to be mediated by cerebellar-specific generation of hydrogen sulfide. However, it remains unclear how D-cysteine and hydrogen sulfide prevent the disease phenotypes of in vitro and in vivo SCA models.

Intracellular protein degradation systems play pivotal roles in neural survival and the maintenance of neural morphology, including axons and dendrites [14]. We have previously demonstrated that the expression of SCA14- and SCA21-causing proteins impairs chaperone-mediated autophagy (CMA), one of the pathways in the autophagy-lysosome protein degradation system [6,15]. In CMA, substrate proteins are detected with the heat shock cognate 70 kDa protein (Hsc70) and are delivered into lysosomes through lysosome-associated membrane protein 2A (LAMP2A) [16]. Micro RNA (miRNA)-mediated knockdown of LAMP2A in cerebellar neurons triggers motor dysfunction, early glial activation, and neurodegeneration, as observed in the SCA mouse models [17]. These findings strongly suggest that a decline in CMA activity is commonly related to the pathogenesis of various SCAs. Therefore, the preventive effect of D-cysteine on SCA is possibly mediated by the activation of CMA and/or microautophagy (mA), in which the substrate proteins are also recognized by Hsc70 and delivered into late endosomes [18]. From these backgrounds, we focused on the relationship between D-cysteine/hydrogen sulfide and CMA/mA in the present study. We investigated the effects of D-cysteine, 3MP, which is an intermediate of the pathway in hydrogen sulfide generation from D-cysteine (Figure 1), and a hydrogen sulfide donor, sodium sulfide (Na_2_S), on CMA and mA using a CMA/mA activity marker that we have previously established [15,19].

## 2. Materials and Methods

### 2.1. Materials

Neuron Culture Medium (#148−09671), Neuron Dissociation Solutions (#297−78001), 3,3′,5′-triiodo-L-thyronine sodium salt (#038-25541), and ScreenFect siRNA (#295−75003) were obtained from FUJIFILM Wako Pure Chemical Industries (Osaka, Japan). Sodium 3-mercaptopyruvate dihydrate (#90374), anti-3MST rabbit polyclonal (#HPA001240), anti-mouse LAMP2 (LAMP2A) rabbit polyclonal (#L0668), and anti-β-actin mouse monoclonal (#A1978) antibodies were obtained from Sigma Aldrich (St. Louis, MO, USA). siRNAs for LAMP2A (sense: 5′-GGCAGGAGUACUUAUUCUAGU-3′, antisense: 5′-UAGAAUAAGUACUCCUGCCAA-3′) and for tumor susceptibility gene 101 protein (TSG101) (sense: 5′-CUAGUUCAAUGACUAUUAATT-3′, antisense: 5′-UUAAUAGUCAUUGAACUAGTT-3′) were obtained from Sigma-Aldrich. Universal negative control siRNA was obtained from Nippon Gene (Tokyo, Japan). Anti-DAO rabbit polyclonal (#GTX53998) antibodies were obtained from GeneTex (Irvine, CA, USA). Anti-human LAMP2A rabbit polyclonal antibody (#ab125068) was obtained from AbCam (Cambridge, United Kingdom). Anti-TSG101 rabbit polyclonal antibody (#sc-7964) was obtained from Santa Cruz Biotechnology (Dallas, TX, USA). Anti-myocyte enhancer factor 2D (MEF2D) mouse monoclonal antibody (#610774) was obtained from BD Biosciences (San Jose, CA, USA). Anti-Hsc70 mouse monoclonal IgM antibody (#NB120-2788) was obtained from Novus Biologicals (Centennial, CO, USA). Anti-nuclear factor-erythroid 2-related factor 2 (Nrf2) rabbit polyclonal antibody (#16396-1-AP) was obtained from ProteinTech (Rosemont, IL, USA). HaloTag ligand fused with tetramethylrhodamine (TMR-HT ligand, #G8251) was obtained from Promega (Madison, WI, USA). D-cysteine hydrochloride monohydrate (#10311−04) and penicillin–streptomycin mixed solution (#26523-84) were obtained from Nacalai Tesque (Kyoto, Japan). Na_2_S (#SB01) was purchased from Dojindo Laboratories (Kumamoto, Japan). Indole-2-carboxylic acid (#I0332), a DAO inhibitor (Figure 1), was purchased from Tokyo Chemical Industry (Tokyo, Japan). 3MST inhibitor (#Z16078832, Figure 1) was purchased from Enamine (Kyiv City, Ukraine) and reported as compound 1 in the manuscript by Hanaoka K et al. [20]. ML385 (#S8798) was obtained from Selleck chemicals (Houston, TX, USA). AD293 cells (#240085), derived from HEK293 cells with improved cell adherence, were purchased from Agilent (Santa Clala, CA, USA).

### 2.2. Cell Culture Experiments Using AD293 Cells

We used AD293 cells that were stably expressing glyceraldehyde 3-phosphate dehydrogenase fused with HaloTag (GAPDH-HT), as described previously [19]. Cells were cultured in Dulbecco’s modified Eagle medium supplemented with 10% fetal bovine serum (FBS), 100 units/mL of penicillin, and 100 mg/mL of streptomycin in a humidified atmosphere with 5% CO_2_ at 37 °C. Cells were spread onto 35 mm glass dishes (#3961-035, AGC Techno Glass, Shizuoka, Japan) coated with poly-L-lysine for the assessment of CMA/mA activity.

For the investigation of the effects of these chemicals on CMA and mA separately, AD293/GAPDH-HT cells (2 × 10^5^ cells/well) were transfected on 24-well plates with 50 pmol negative control siRNA or siRNA for LAMP2A, a CMA-related protein, or siRNA for TSG101, an mA-related protein using ScreenFect siRNA according to the manufacturer’s protocol. After cultivation for 4 h, cells were detached and spread onto both glass dishes.

Cells on the glass dishes were incubated with 50 nM TMR-HT ligand for 10 min at 37 °C for labeling of GAPDH-HT with TMR. Then, cells were cultured in control media or media containing D-cysteine (0.5 or 1 mM), 3MP (0.2 or 0.5 mM), or Na_2_S (10 μM) for 24 h, followed by fixation in 4% paraformaldehyde (PFA). In several experiments, cells were treated with these chemicals in the presence of a vehicle (0.1% dimethyl sulfoxide), 3MST inhibitor (10 μM), or ML385 (0.1 or 1 μM), an Nrf2 inhibitor. We observed TMR fluorescence of GAPDH-HT in cells using a confocal laser microscope (TCS-SP5, Leica Microsystems, Wetzlar, Germany). The CMA/mA activity in each cell was quantified as the number of GAPDH-HT puncta per cell. GAPDH-HT puncta in cells under LAMP2A and TSG101 knockdown reflect mA and CMA activities, respectively [19]. GAPDH-HT puncta were manually counted using ImageJ software (National Institute of Health, Bethesda, ND, USA). Image acquisition and the counting of GAPDH puncta were blindly conducted by a different experimenter from the one who handled and treated cells with chemicals.

For immunoblot analyses, cells were spread onto 12-well plates (2 × 10^5^ cells/well). After 24 h, cells were cultured with normal media or media containing 10 μM Na_2_S for 4 or 24 h and were lysed in radioimmunoprecipitation assay (RIPA) buffer, as described previously [21].

### 2.3. Experiments Using Primary Cerebellar Cultures

We used six pregnant Wistar rats for the present study. Immediately after the euthanasia by intraperitoneal injection with pentobarbital (100 mg/kg), we isolated cerebella from E16 embryos and prepared the primary cerebellar cultures, as described previously [6]. To assess CMA/mA activity, we added two adeno-associated virus serotype 9 (AAV9) vectors to express GFP under PC-specific L7 promoter and GAPDH-HT under neuron-specific synapsin I promoter to primary cerebellar cultures on day one in vitro (DIV1). AAV9 vectors were prepared using the ultracentrifugation method described in a previous paper [22]. Cultures were incubated with 50 nM of TMR-HT ligand for 10 min at 37 °C on DIV20, followed by procedures similar to those for AD293.

For the assessment of PC morphology, cultures were added with AAV9 vectors to express GFP under the L7 promoter on DIV1 and treated with the normal media or media containing 1 mM D-cysteine or 10 μM Na_2_S on DIV14 and DIV 19, followed by fixation in 4% PFA on DIV24. PC morphology was evaluated from the GFP fluorescence images obtained using a confocal microscope (TCS SP5). The lengths of the longest PC dendrites were manually measured using free software for TCS SP5 (LAS AF lite, Leica Microsystems). We manually traced the dendrites from their origin (the edge of the soma) to the distal ends of the branches. We measured the length of three or four dendritic branches per PC and selected the length of the longest one. Image acquisition and measurement of the dendritic lengths were blindly conducted by a different experimenter than the one who handled and treated the cells with chemicals.

### 2.4. Long-Term Treatment with D-Cysteine in Mice

Ten-week-old male ICR mice were intraperitoneally administered saline (0.9% sodium chloride) or 100 mg/kg D-cysteine hydrochloride daily for 10 weeks. We used ten ICR mice (five for treatment with saline and five for treatment with D-cysteine). After euthanasia by intraperitoneal injection with pentobarbital (100 mg/kg), mouse cerebella were isolated, homogenized, and lysed in RIPA buffer, as previously described [7].

### 2.5. Immunoblot Analyses

Immunoblot experiments were conducted as previously described [7]. Equal amounts of proteins from the cell and cerebellar lysates were subjected to SDS-PAGE, followed by immunoblot analyses using anti-DAO (1:1000), anti-3MST (1:500), human LAMP2A (1:2000), anti-TSG101 (1:1000), anti-mouse LAMP2A (1:2000), anti-Hsc70 (1:2000), anti-Nrf2 (1:1000), anti-MEF2D (1:2000), and anti-β-actin (1:5000) antibodies as primary antibodies. BlueStar Prestained Protein Ladder (#NE-MWP03, Nippon Genetics, Tokyo, Japan) was used for molecular weight markers. Immunoreactive bands were quantified using ImageJ software. Protein amounts were normalized by relative amounts to β-actin.

### 2.6. Statistical Analyses

All quantitative data are presented as bar graphs indicating mean ± standard error of the mean. Data from immunoblot experiments are presented as bar graphs with individual data points. Statistical differences for two groups were determined with unpaired Student’s *t*-tests using Microsoft Excel. Statistical differences for more than three groups were determined with one-way analysis of variance (ANOVA) followed by a post-hoc Tukey test or with Kruskal–Wallis tests followed by post-hoc Dunn’s tests for more than three groups using the GraphPad Prism 6 software (GraphPad Software, San Diego, CA, USA). Probability values of less than 0.05 were considered significant.

## 3. Results

### 3.1. Effects of D-Cysteine, 3MP, and Na_2_S on CMA/Ma Activity in Primary Cultured Purkinje Cells and AD293 Cells

**Figure 2:**
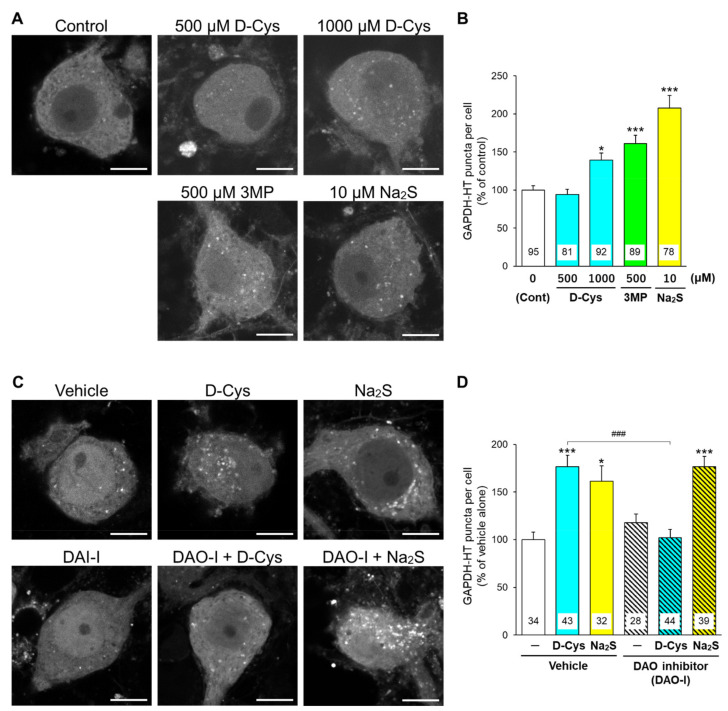
Effects of D-cysteine, 3MP, and Na_2_S on CMA/mA activity in primary cultured PCs. (**A**) Punctate accumulation of GAPDH-HT in primary cultured PCs treated with D-cysteine (D-Cys, 500 and 1000 μM), 3MP (500 μM), and Na_2_S (10 μM) for 24 h. Control (Cont) refers to cells without treatment. (**B**) Quantitative analyses of GAPDH-HT puncta shown in A. (**C**) Punctate accumulation of GAPDH-HT in AD293 cells treated with D-cysteine (1000 μM) and Na_2_S (10 μM) in the presence of vehicle (0.1% dimethyl sulfoxide) or DAO inhibitor (DAO-I, 5 μM) for 24 h. (**D**) Quantitative analyses of GAPDH-HT puncta are shown in C. Scale bars = 10 μm. Numbers in the columns represent the numbers of observed cells. * *p* < 0.05, *** *p* < 0.001 vs. control or vehicle alone, ### *p* < 0.001 (Dunn’s multiple comparisons test).

**Figure 3:**
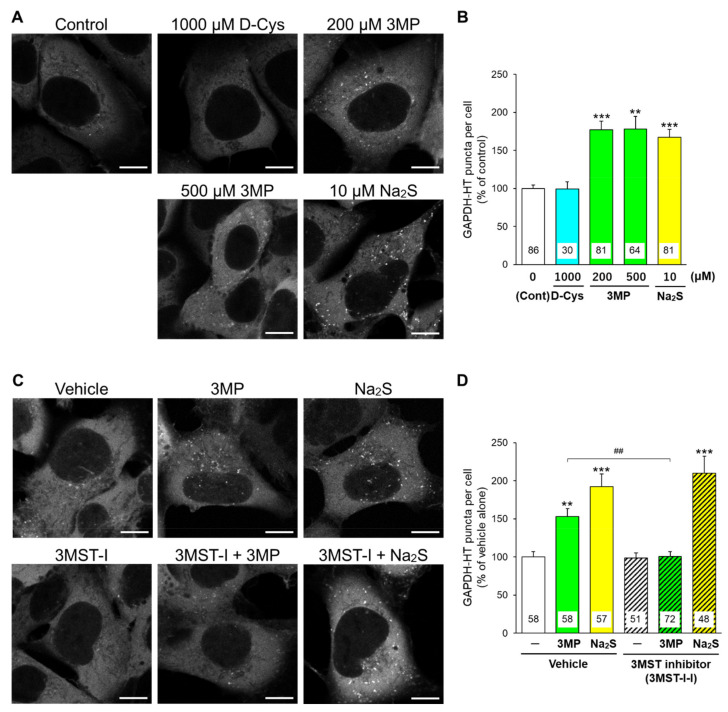
Effects of D-cysteine, 3MP, and Na_2_S on CMA/mA activity in AD293 cells stably expressing GAPDH-HT. (**A**) Punctate accumulation of GAPDH-HT in AD293 cells treated with D-cysteine (1000 μM), 3MP (200 and 500 μM), and Na_2_S (10 μM) for 24 h. Control refers to cells without treatment. (**B**) Quantitative analyses of GAPDH-HT puncta shown in A. (**C**) Punctate accumulation of GAPDH-HT in AD293 cells treated with 3MP (200 μM) and Na_2_S (10 μM) in the presence of a vehicle (0.1% dimethyl sulfoxide or 3MST inhibitor (3MST-I, 100 μM) for 24 h. (**D**) Quantitative analyses of GAPDH-HT puncta shown in C. Scale bars = 10 μm. Numbers in the columns represent the numbers of observed cells. ** *p* < 0.01, *** *p* < 0.001 vs. control or vehicle alone, ## *p* < 0.01 (Dunn’s multiple comparisons test).

Glyceraldehyde 3-phosphate dehydrogenase (GAPDH) is recognized by Hsc70 and is a common substrate for CMA and mA [18]. We used GAPDH fused with HaloTag (GAPDH-HT) as a marker for evaluating the CMA/mA activity [15,19]. We have previously demonstrated that the punctate accumulation of GAPDH-HT reflects CMA/mA activity in cells. Immunoblot experiments revealed that DAO and 3MST were expressed in primary cerebellar cultures, while AD293 cells expressed 3MST but not DAO (Figure S1). Therefore, we compared the effects of D-cysteine, 3MP, and Na_2_S on CMA/mA activity between DAO-expressing cerebellar primary cultures and DAO-deficient AD293 cells. In primary cultured PCs transiently expressing GAPDH-HT, D-cysteine (1000 μM (1 mM)), 3MP (500 μM (0.5 mM)), and Na_2_S (10 μM) significantly increased the punctate accumulation of GAPDH-HT (Figure 2A,B). An inhibitor of DAO (indole-2-carboxylic acid, Figure 1) [10] significantly inhibited the increase in GAPDH-HT puncta by D-cysteine in PCs, while it did not hamper the Na_2_S-mediated increase (Figure 2C,D). In AD293 cells stably expressing GAPDH-HT, D-cysteine (1000 μM (1 mM)) failed to increase the punctate accumulation of GAPDH-HT, while 3MP (200 and 500 μM [0.2 and 0.5 mM]) and Na_2_S (10 μM) significantly increased it (Figure 3A,B). An inhibitor of 3MST (Figure 1) [20] significantly reversed the effect of 3MP on the punctate accumulation of GAPDH-HT in AD293 cells. In contrast, this inhibitor did not affect the increase in GAPDH-HT puncta by Na_2_S (Figure 3C,D). The results indicated in Figure 2 and Figure 3 suggest that generation of hydrogen sulfide from D-cysteine and 3MP is essential for CMA and/or mA activation.

**Figure 4:**
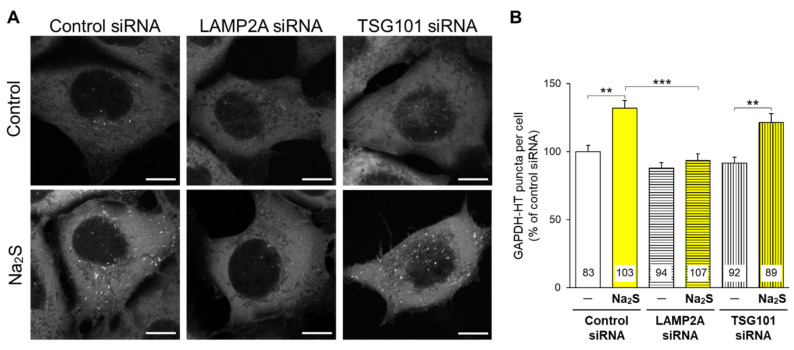
Separate assessment of CMA and mA activities in response to Na_2_S in AD293 cells. (**A**) Punctate accumulation of GAPDH-HT in AD293 cells transfected with control, LAMP2A, or TSG101 siRNA, followed by the treatment with Na_2_S (10 μM) for 24 h. Scale bars are 10 μm. siRNA-mediated knockdown of LAMP2A and TSG101 was confirmed by immunoblotting (Figure S2). (**B**) Quantitative analyses of GAPDH-HT puncta shown in A. Numbers in the columns represent the numbers of observed cells. ** *p* < 0.01, *** *p* < 0.001 (Dunn’s multiple comparisons test).

Next, we attempted to determine the main pathway (CMA or mA) that is activated by hydrogen sulfide. We previously demonstrated that CMA and mA activities are separately evaluated in cells with siRNA-mediated knockdown of an mA-related protein, TSG101, or a CMA-related protein, LAMP2A, respectively [19]. Na_2_S significantly elevated the punctate accumulation of GAPDH-HT in AD293 cells transfected with negative control siRNA or TSG101 siRNA, while it failed to increase the accumulation in LAMP2A-knockdown cells (Figure 4). This result suggests that hydrogen sulfide mainly activates CMA but does not affect mA activity.

### 3.2. Involvement of Nrf2 in D-Cysteine—And Na_2_S-Induced Activation of CMA in Primary Cultured Purkinje Cells and AD293 Cells

**Figure 5:**
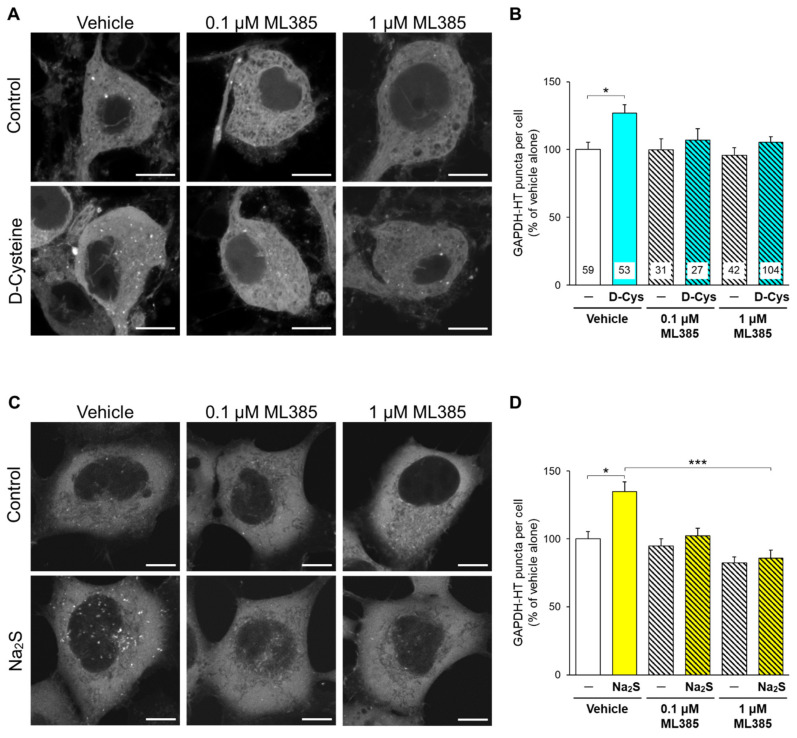
Effect of an Nrf2 inhibitor on the D-cysteine- and Na_2_S-induced activation of CMA in primary cultured PCs and AD293 cells. (**A**) Punctate accumulation of GAPDH-HT in primary cultured PCs treated with D-cysteine (1 mM) in the presence of a vehicle (0.1% dimethyl sulfoxide) or ML385 (0.1 and 1 μM) for 24 h. (**B**) Quantitative analyses of GAPDH-HT puncta shown in A. (**C**) Punctate accumulation of GAPDH-HT in AD293 cells treated with Na_2_S (10 μM) in the presence of the vehicle or ML385 for 24 h. (**D**) Quantitative analyses of GAPDH-HT puncta shown in C. Scale bars = 10 μm. Numbers in the columns represent the numbers of observed cells. * *p* < 0.05, *** *p* < 0.001 (Dunn’s multiple comparisons test).

We then explored the mechanism of how hydrogen sulfide activates CMA. The activation of nuclear factor erythroid 2-related factor 2 (Nrf2) upregulates LAMP2A, leading to CMA activation [23]. Hydrogen sulfide is reported to activate Nrf2 [24]. In the normal condition, Nrf2 is ubiquitinated by kelch-like ECH-associated protein 1 (Keap1) and degraded via proteasome [25]. Hydrogen sulfide induces the cysteine polysulfidation of Keap1 and dissociates Nrf2 from Keap1, leading to the upregulation and activation of Nrf2 [24]. Therefore, we investigated the involvement of Nrf2 in CMA activation. ML385, an inhibitor of Nrf2 [26], significantly hampered the increase in punctate accumulation of GAPDH-HT by D-cysteine in PCs (Figure 5A,B) and by Na_2_S in AD293 cells (Figure 5C,D).

**Figure 6:**
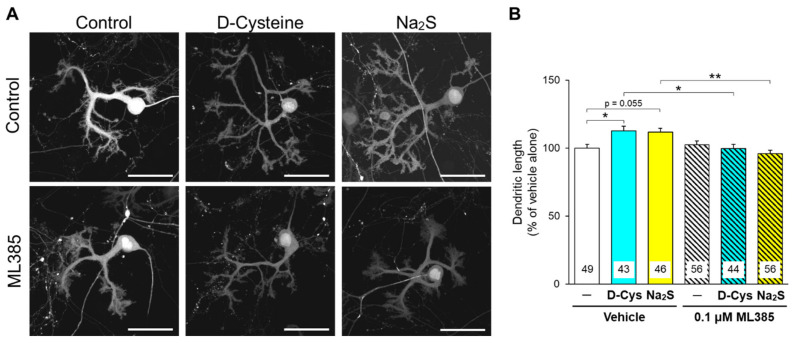
Effect of an Nrf2 inhibitor on the D-cysteine- and Na_2_S-induced enhancement of dendritic development in primary cultured PCs. (**A**) Fluorescent images of GFP-expressing PCs treated with D-cysteine (1 mM) and Na_2_S (10 μM) in the presence of a vehicle (0.1% dimethyl sulfoxide) or ML385 (0.1 μM) for 10 days. Scale bars are 50 μm. (**B**) Quantitative analyses of the dendritic length of PCs shown in A. Numbers in the columns represent the numbers of observed cells. * *p* < 0.05, ** *p* < 0.01 (Turkey’s multiple comparisons test).

Similar to the results of our previous report [13], D-cysteine significantly enhanced the dendritic development of primary cultured PCs, and Na_2_S tended to enhance it (Figure 6). ML385 (0.1 μM) significantly prevented this enhancement via D-cysteine and Na_2_S (Figure 6), suggesting that Nrf2 is involved in the hydrogen sulfide-mediated enhancement of dendritic development in PCs.

### 3.3. Effects of D-Cysteine and Na_2_S on the Expression of Nrf2 and CMA-Related Proteins in AD293 Cells and Mouse Cerebellum

**Figure 7:**
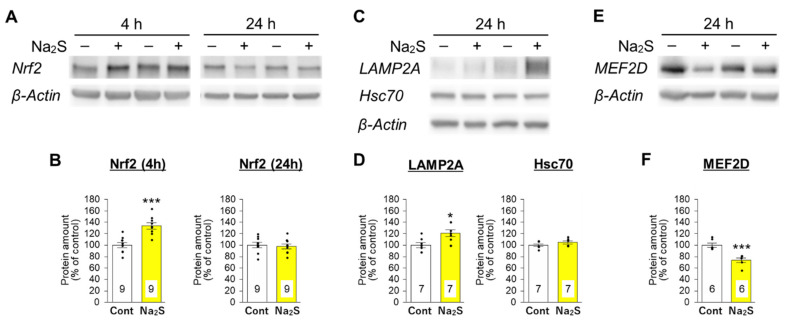
Effect of Na_2_S on the amounts of Nrf2 and CMA-related proteins in AD293 cells. (**A**) Immunoblot analyses of Nrf2 and β-actin in cell lysates from AD293 cells treated with Na_2_S (10 μM) for 4 and 24 h. (**B**) Quantitative analyses of Nrf2 amounts from the immunoblot results shown in A. (**C**) Immunoblot analyses of CMA-related proteins (LAMP2A, Hsc70) and β-actin in cell lysates from AD293 cells treated with Na_2_S (10 μM) for 24 h. (**D**) Quantitative analyses of LAMP2A and Hsc70 amounts from the immunoblot results shown in C. (**E**) Immunoblot analyses of a CMA/mA substrate (MEF2D) and β-actin in cell lysates from AD293 cells treated with Na_2_S (10 μM) for 24 h. (**F**) Quantitative analysis of MEF2D amount from the immunoblot results shown in F. Whole blot images are presented in Figure S5. Amounts of β-actin were used as internal controls for quantification. Numbers in the columns represent the number of samples. * *p* < 0.05, *** *p* < 0.001 (unpaired *t*-test).

Next, we investigated whether hydrogen sulfide activates Nrf2 by immunoblotting cellular lysates from AD293 cells. Nrf2 was significantly increased 4 h after Na_2_S stimulation, whereas Na_2_S did not affect the amount of Nrf2 24 h after the treatment (Figure 7A,B). These findings suggest that hydrogen sulfide transiently increases Nrf2 immediately after stimulation. Thereafter, an increase in LAMP2A and a decrease in myocyte enhancer factor 2D (MEF2D), a CMA/mA substrate [27], were observed in cell lysates from AD293 cells 24 h after Na_2_S treatment (Figure 7C–F). In contrast, Na_2_S treatment did not affect the amount of Hsc70 (Figure 7C,D). Furthermore, the Na_2_S-mediated changes in LAMP2A and MEF2D were blocked by the co-treatment with ML385 (Figure S3). These results suggest that CMA activation is followed by the rapid activation of Nrf2 by hydrogen sulfide.

**Figure 8:**
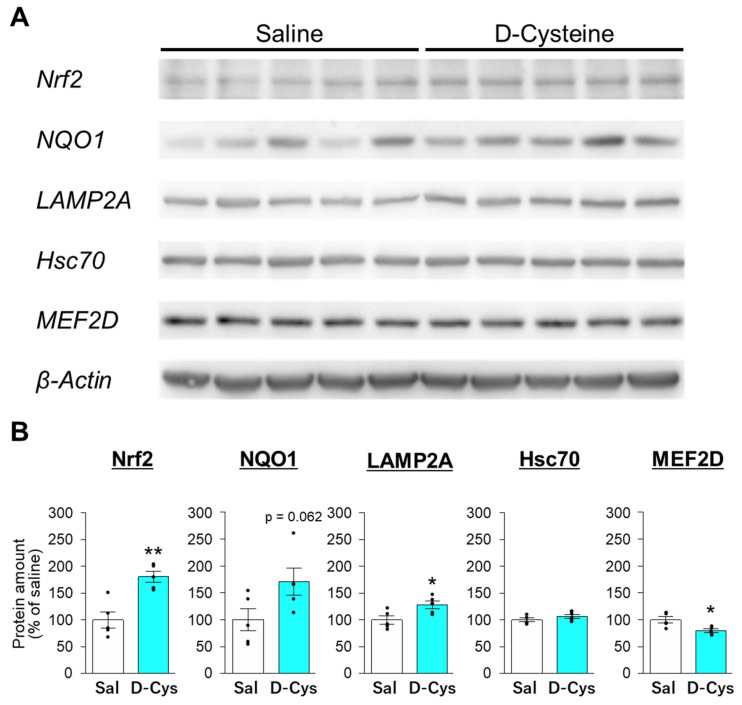
Effect of long-term treatment with D-cysteine on the amounts of Nrf2- and CMA-related proteins in cerebellar lysates from ICR mice. (**A**) Immunoblot analyses of Nrf2, NQO1, LAMP2A, Hsc70, MEF2D, and β-actin in cerebellar lysates from ICR mice daily treated with saline (Sal) and D-cysteine (100 mg/kg/day) for 10 weeks. Whole blot images are presented in Figure S6. (**B**) Quantitative analyses of the amounts of Nrf2, NQO1, LAMP2A, Hsc70, and MEF2D shown in A. Amounts of β-actin were used as internal controls for the quantification. * *p* < 0.05, ** *p* < 0.01 vs. saline-treated mice (unpaired *t*-test, *n* = 5 in both saline- and D-cysteine-treated mice).

Finally, we investigated the effect of long-term administration of D-cysteine on the expression of Nrf2- and CMA-related proteins in mouse cerebella. ICR mice were administered with 100 μg/kg D-cysteine or saline once every 24 h for 10 weeks. D-Cysteine significantly increased the amount of Nrf2 and tended to increase the amount of NAD(P)H quinone dehydrogenase 1 (NQO1), a downstream protein of the Nrf2 pathway [25] (Figure 8), suggesting that long-term treatment of D-cysteine activates Nrf2 in mouse cerebella. D-cysteine significantly increased LAMP2A in cerebellar lysates, whereas it did not affect the amount of Hsc70 (Figure 8). Moreover, D-cysteine significantly decreased the amount of MEF2D, a substrate for CMA/mA, in cerebellar lysates (Figure 8). In contrast, D-cysteine did not significantly affect the amounts of Nrf2, LAMP2A, and MEF2D in lysates from the cerebral cortices (Figure S4). These findings indicate that D-cysteine enhances CMA activity via Nrf2 activation in the cerebellum, but not in the cerebral cortex.

## 4. Discussion

Intracellular protein degradation systems are crucial for maintaining intracellular protein homeostasis, especially in the neurons [14]. The autophagy–lysosome system is one of the main intracellular protein degradation systems and comprises three pathways, macroautophagy (MA), mA, and CMA [28]. Among these, MA is the most widely studied [29,30]. Neuron-selective deficiency of MA induces accumulation of unnecessary proteins and neurodegeneration, which are frequently observed in models of neurodegenerative diseases [31,32]. Therefore, MA impairment is considered essential for the etiology of neurodegenerative diseases. In contrast, excessive MA is reported to contribute to the ischemic brain injury [33]. Therefore, the role of MA in neuronal survival is still unclear. Hydrogen sulfide has a neuroprotective activity [34]. Although several reports have demonstrated the involvement of MA in hydrogen sulfide-mediated neuroprotection, the effects of hydrogen sulfide on MA are also controversial [35,36]. Several studies have revealed that hydrogen sulfide exerts neuroprotective effects via the activation of MA [37,38], while MA inhibition contributes to the hydrogen sulfide-mediated neuroprotection [39,40].

In the present study, we first revealed that hydrogen sulfide activates CMA. Compared with MA, CMA and mA have been studied less [28]. Hsc70 is commonly involved in CMA and mA by recognizing and delivering substrates [16,18]. Hsc70 recognizes substrate proteins via a pentapeptide sequence called the KFERQ motif. Since approximately 75% of the total proteins in the human proteome have the KFERQ motifs [41], CMA and mA are also crucial for maintaining protein homeostasis. CMA impairment is potentially involved in the pathogenesis of various neurodegenerative diseases [42], as well as SCA, which we described in the introduction. Notably, CMA impairment is observed in the tissues from patients with Parkinson’s and Alzheimer’s diseases [43,44]. Furthermore, neurodegeneration is triggered in the dopaminergic neurons of the substantia nigra by the CMA impairment that is induced by shRNA-mediated knockdown of LAMP2A [45]. Conversely, LAMP2A overexpression attenuates neurodegeneration in cellular and animal models of Parkinson’s disease [46]. Moreover, the chemicals that activate CMA have therapeutic effects in cellular and animal models of Alzheimer’s disease [44,47]. These findings suggest that CMA can be a potential target for the treatment of various neurodegenerative diseases, including SCA, Parkinson’s disease, and Alzheimer’s disease. Since CMA activation might aid in the neuroprotective effect of hydrogen sulfide, it can provide a useful approach for the treatment of various neurodegenerative diseases.

The treatment with Na_2_S rapidly increased the amount of Nrf2 in AD293 cells. However, this effect did not last 24 h after the treatment (Figure 7). Since release of hydrogen sulfide immediately follows the treatment with Na_2_S, it can rapidly modify the cysteine residues of Keap1, leading to the dissociation of Nrf2 from Keap1 and subsequent Nrf2 elevation and activation [24]. This result is consistent with our previous finding that chemicals that directly interact with Keap1 immediately increase the amount of Nrf2 [48]. Activation of Nrf2 leads to upregulation of various antioxidative proteins, including NQO1 and heme oxygenase-1 (HO-1) [25]. LAMP2A is also upregulated by of Nrf2 activation [23]. In the present study, we observed an increase in LAMP2A and decrease in MEF2D, a substrate for CMA/mA, in Na_2_S-treated AD293 cells and D-cysteine-treated cerebellar lysates (Figure 7 and Figure 8). Taken together with the finding that ML385, an Nrf2 inhibitor, suppresses the CMA-activation triggered by D-cysteine and Na_2_S (Figure 5), Nrf2 activation is understood to be involved in hydrogen sulfide-mediated activation of CMA.

In the present study, we revealed that in vivo long-term treatment with D-cysteine activates Nrf2 in the mouse cerebellum (Figure 8). This finding indicates that D-cysteine-induced activation of Nrf2 is possibly involved in D-cysteine-mediated preventive effect on SCA, as demonstrated previously [7]. Lee et al. (2014) revealed that Nrf2-target proteins, NQO1 and HO-1, are downregulated in lymphoblastoid cells of SCA17 [49], indicating decreased activity of Nrf2. Several reports have revealed that a decrease in Nrf2 activity is observed in model cells of Friedrich ataxia and ataxia telangiectasia, both of which are autosomal recessive ataxias [50,51]. Furthermore, chemicals that activate Nrf2 mitigate disease phenotypes in cellular and Drosophila models of SCA3 [52]. These findings raise the possibility that Nrf2 impairment commonly participates in the pathogenesis of cerebellar ataxia. Since Hsc70 is also downregulated in lymphoblastoid cells of SCA17 [49], CMA activity can also be impaired in these cells. Based on our previous findings [6,15,17], we proposed that the decrease in CMA activity is related to pathogenesis of various SCAs. The reduced activity of Nrf2 might cause this CMA impairment. Further studies are necessary to reveal the role of Nrf2 in the pathogenesis of SCAs.

Several D-amino acids are endogenously generated in mammals and have roles different from those of L-amino acids [53]. Among D-amino acids, D-serine is most widely studied for its functions in the brain [54]. D-serine is endogenously generated from L-serine by serine racemase and is degraded by DAO [11]. D-serine works as a co-agonist of N-methyl-D-aspartic acid (NMDA) receptor, and its reduction is related to the pathogenesis of schizophrenia [55]. Additionally, D-serine is an endogenous agonist of an orphan type glutamate receptor, GluRδ2 [56], which is selectively expressed in cerebellar PCs. On the other hand, the presence of endogenous D-cysteine had not been determined in mammals. A recent report indicated that D-cysteine is endogenously generated from L-cysteine by serine racemase [57]. Although endogenous D-cysteine is most abundant at the embryonic stage and enhances the differentiation of neural progenitor cells to neurons, it is also detected in mouse brain lysates and human cerebrospinal fluids. Semenza et al. (2021) also revealed that D-cysteine directly interacts with myristoylated alanine-rich c-kinase substrate (MARCKS), which is a membrane-anchored protein and translocates to the cytoplasm in response to phosphorylation by protein kinase C [58] and inhibits its phosphorylation and translocation in neural progenitor cells [57]. Therefore, it is possible that D-cysteine activates Nrf2 through MARCKS. In the present study, D-cysteine activated Nrf2 in the cerebellum but not in the cerebral cortex (Figure 8, Figure S4). Since MARCKS is distributed in various brain regions, including the cerebral cortex [59], it is not possible that MARCKS is involved in the D-cysteine-mediated activation of Nrf2. Since DAO is expressed in the cerebellum but not in the cerebral cortex [10], DAO-mediated hydrogen sulfide production from D-cysteine would confer the cerebellar-selective activation of Nrf2 and CMA.

In addition to the cerebellum, DAO is also expressed in the liver and kidney [11,12]. Therefore, D-cysteine can also work as a hydrogen sulfide donor for the liver and kidneys. Since oxidative stress is commonly involved in various chronic diseases, Nrf2 could be a therapeutic target for these chronic diseases [60]. Moreover, CMA failure has also been reported in patients with chronic liver and kidney diseases [61,62]. It is expected that D-cysteine can be used as a novel Nrf2 and CMA activator selective for the cerebellum, liver, and kidneys via hydrogen sulfide production.

## 5. Conclusions

Our study suggests that D-cysteine activates CMA in cerebellar PCs via the generation of hydrogen sulfide and the activation of Nrf2. Since several findings indicate that CMA impairment is related to SCA pathogenesis [6,15,17], this CMA-activating property of D-cysteine might contribute to its therapeutic effect on in vitro and in vivo SCA models [7]. In contrast, several reports indicate that Nrf2 activation mediates the anti-inflammatory effect [63,64]. Therefore, the Nrf2-mediated anti-inflammatory effect might be related to the protective effect of D-cysteine on SCA model mice. Although further studies are necessary to elucidate the involvement of CMA in the protective effect of D-cysteine, the present findings provide the possible therapeutic mechanism of D-cysteine and the availability of D-cysteine for SCA and related cerebellar diseases.

## Data Availability

The data presented in this study are available on request from the corresponding author.

## Supplementary Materials

The following are available online at https://www.mdpi.com/article/10.3390/cells11071230/s1, Figure S1: Expression of DAO and 3MST in AD293 cells and primary cerebellar cultures. Figure S2: Confirmation of siRNA-mediated knockdown of LAMP2A and TSG101 in AD293 cells by immunoblotting. Figure S3: Effect of ML385 on the Na2S-mediated changes in the amounts of LAMP2A and MEF2D in AD293 cells. Figure S4: Effect of long-term treatment with D-cysteine on the amounts of Nrf2- and CMA-related proteins in lysates from cerebral cortices of ICR mice. Figure S5: Whole blot images of immunoblot experiments in Figure 7, Figure S6: Whole blot images of immunoblot experiments in Figure 8.
