# Supplementary material for: D-Cysteine Activates Chaperone-Mediated Autophagy in Cerebellar Purkinje Cells via the Generation of Hydrogen Sulfide and Nrf2 Activation
Source: Cells. 2022 Apr 5;11(7):1230. doi: 10.3390/cells11071230 (PMC8997644; doi:10.3390/cells11071230)
Supplement: Supplementary file 1 [file cells-11-01230-s001.zip › cells-1645351-supplementary.pdf]

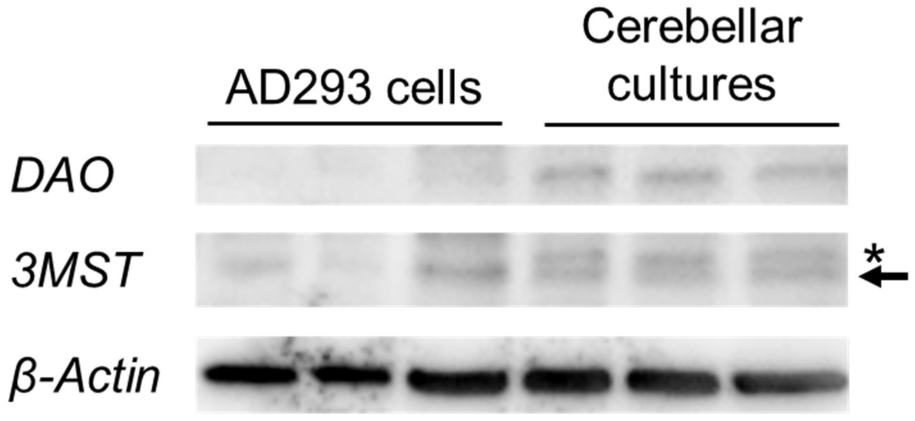

Figure S1. Expression of DAO and 3MST in AD293 cells and primary cerebellar cultures. The same blot was detected with anti-DAO antibody, followed by the sequential detection with anti-3MST antibody and anti-β-actin antibody. Arrow indicates the 3MST bands. Asterisk indicates the residual bands detected with anti-DAO antibody.

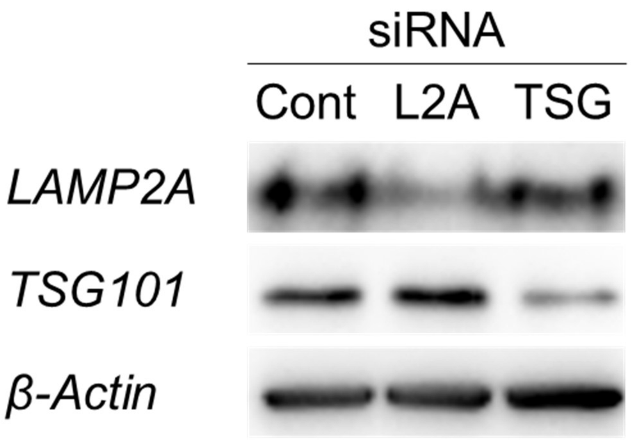

Figure S2. Confirmation of siRNA-mediated knockdown of LAMP2A and TSG101 in AD293 cells by immunoblotting. We present representative immunoblots of lysates from cells transfected with control (Cont), LAMP2A (L2A), and TSG101 (TSG) siRNA, detected with anti-LAMP2A, anti-TSG101, and anti-β-actin antibodies.

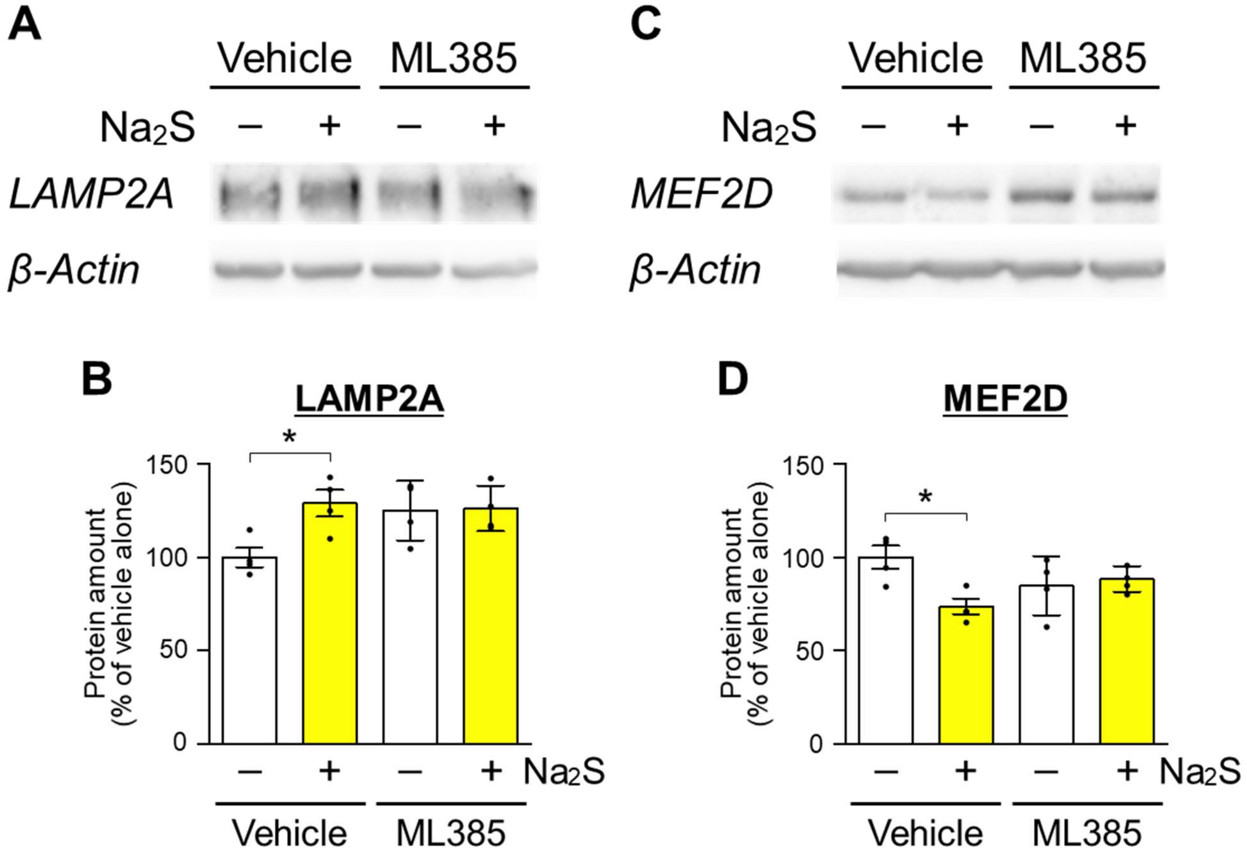

Figure S3. Effect of ML385 on the Na_2S-mediated changes in the amounts of LAMP2A and MEF2D in AD293 cells. (A) Immunoblot analyses of LAMP2A and β-actin in cell lysates from AD293 cells treated with Na_2S (10 μM) in the presence a vehicle (0.1 % dimethyl sulfoxide) or ML385 (0.1 μM) for 24 h. (B) Quantitative analyses of the amount of LAMP2A shown in A. Amounts of β-actin were used as internal controls for the quantification. Although ML385 tended to increase LAMP2A, a significant difference was observed only between control and Na_2S-treated cells in the presence of a vehicle (* $p < 0.05$, Tukey's multiple comparisons test, n = 4). (C) Immunoblot analyses of MEF2D and β-actin in cell lysates from AD293 cells treated with Na_2S (10 μM) in the presence a vehicle (0.1 % dimethyl sulfoxide) or ML385 (0.1 μM) for 24 h. (D) Quantitative analyses of the amount of LAMP2A shown in C. Amounts of β-actin were used as internal controls for the quantification. A significant difference was observed only between control and Na_2S-treated cells in the presence of a vehicle (* $p < 0.05$, Tukey's multiple comparisons test, n = 4).

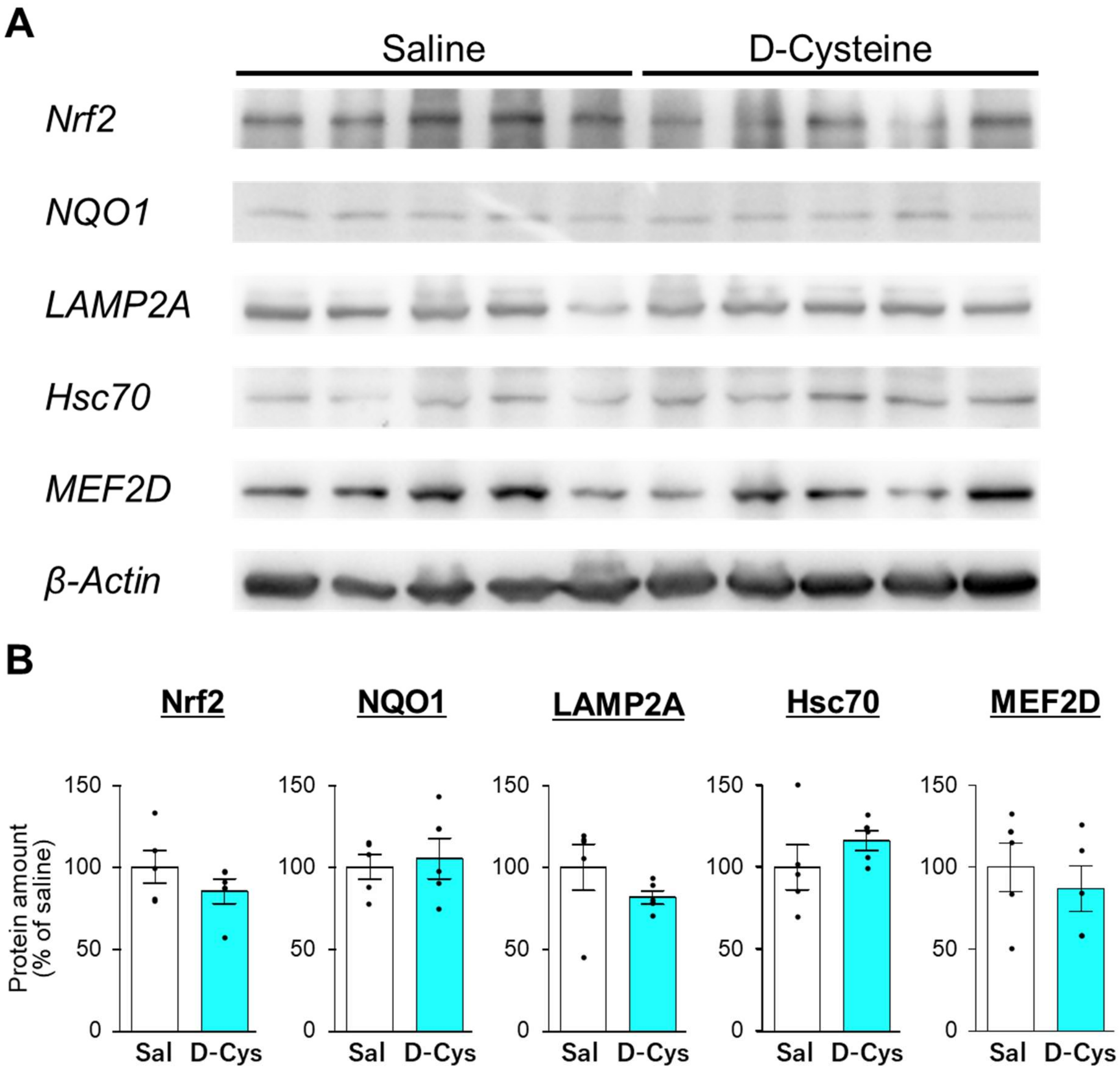

Figure S4. Effect of long-term treatment with D-cysteine on the amounts of Nrf2- and CMA-related proteins in lysates from cerebral cortices of ICR mice. (**A**) Immunoblot analyses of Nrf2, NQO1, LAMP2A, Hsc70, MEF2D, and β-actin in cerebellar lysates from ICR mice daily treated with saline (Sal) and D-cysteine (100 mg/kg/day) for 10 weeks. (**B**) Quantitative analyses of the amounts of Nrf2, NQO1, LAMP2A, Hsc70, and MEF2D shown in A. Amounts of β-actin were used as internal controls for the quantification. There were no significant differences in the amounts of all examined proteins between saline- and D-cysteine-treated mice (unpaired t-test, n = 5).

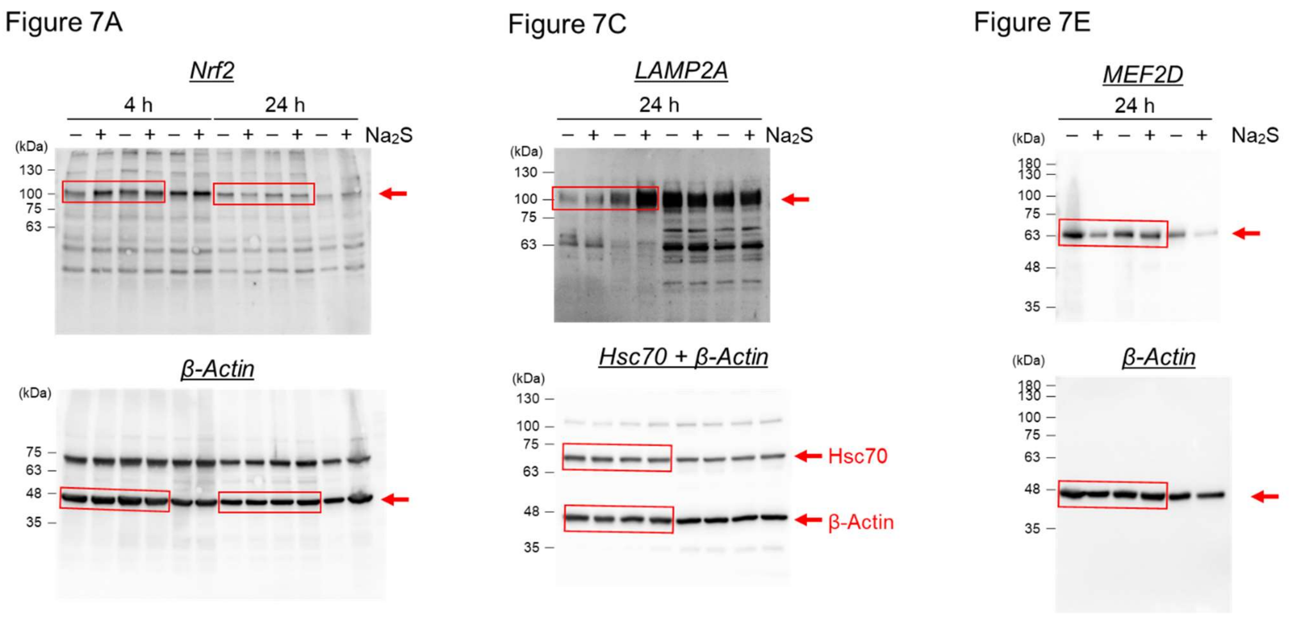

Figure S5. Whole blot images of immunoblot experiments in figure 7. Red arrows indicate the bands of target proteins. Red rectangles indicate the cropped positions of the blots that are shown in Figure 7.

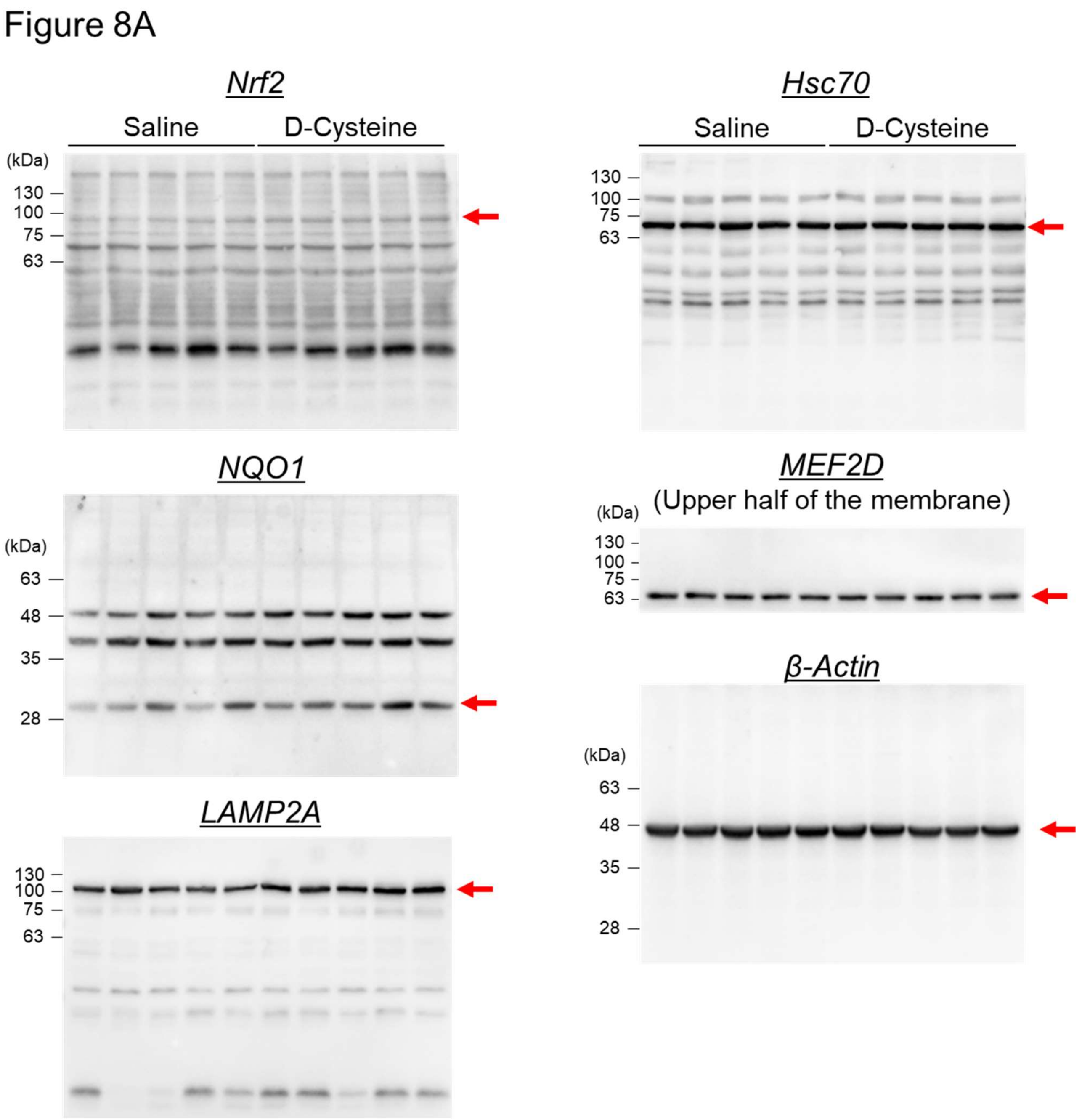

Figure S6. Whole blot images of immunoblot experiments in figure 8A. Red arrows indicate the bands of target proteins.
